# Investigating Raman peak enhancement in carboxyl-rich molecules: insights from Au@Ag core-shell nanoparticles in colloids

**DOI:** 10.3389/fchem.2025.1522043

**Published:** 2025-01-27

**Authors:** Junhao Chen, Zhengjia Chen, Tong Liang, Zhennan Zhang, Dahang Cheng, Shurui Liu, Haiyang Liu, Cuicui Liu, Xiaohui Song

**Affiliations:** ^1^ School of Materials Science and Engineering, Hefei University of Technology, Hefei, Anhui, China; ^2^ General Education and International School, Chongqing Polytechnic University of Electronic Technology, Chongqing, China; ^3^ Department of Chemistry and Biochemistry, Nanyang Technological University, Singapore, Singapore

**Keywords:** surface-enhanced Raman scattering, Au@Ag core-shell nanoparticles, citric acid, carboxyl-rich molecule, colloidal

## Abstract

Surface-enhanced Raman scattering (SERS) is a promising analytical technique with applications in environmental monitoring, healthcare, and the biopharmaceutical industry. While SERS has been successfully applied to molecules such as 4-mercaptobenzoic acid and other thiol- and amine-containing compounds, there is limited research on its detection capabilities for molecules rich in carboxyl groups or unsaturated bonds, such as citric acid. This study investigates the SERS enhancement of Au@Ag core-shell nanoparticles in response to citric acid and other molecules with carboxyl and unsaturated bonds. We compare the SERS behavior of nanoparticles in freshly prepared and aged sodium citrate solutions to identify differences in Raman peak enhancement. Our findings show that the Au@Ag core-shell nanoparticles exhibit significant SERS enhancement when exposed to citric acid and other related compounds. The enhancement varies based on the age of the sodium citrate solution, which influences the structural properties of the nanoparticles. This work opens avenues for further research and applications in biological monitoring, environmental testing, and the pharmaceutical industry.

## 1 Introduction

Surface-enhanced Raman scattering (SERS) has emerged as a powerful analytical technique, providing highly sensitive and selective detection of various molecules (Bhattacharjee et al., 2018a). The method exploits the enhancement of Raman signals through the interaction of molecules with metallic nanostructures, particularly gold and silver, which can produce significant amplification effects (Wu et al., 2012; Yilmaz and Yilmaz, 2020). The enhancement mechanisms in SERS are generally attributed to two complementary effects: electromagnetic (EM) enhancement and chemical (CM) enhancement. The EM mechanism arises from localized surface plasmon resonances (LSPR) in metallic nanostructures, which create intense electromagnetic fields near the nanoparticle surfaces, dramatically boosting Raman scattering signals (Brosseau et al., 2023). Meanwhile, CM enhancement is associated with charge transfer interactions between the molecule and the metal surface, further amplifying signal intensity (Han et al., 2022). Over the past few decades, extensive research has focused on conventional molecules rich in thiol (-SH) and amine (-NH2) functional groups, which readily adsorb onto metal surfaces due to strong chemical bonds (Morovvati and Malekfar, 2019; Patra and Kumar, 2013). Notable examples include 4-mercaptobenzoic acid (4-MBA) and cysteine, both of which have been extensively studied for their SERS activity, demonstrating high sensitivity and reproducibility (Sugawa et al., 2013; Guo et al., 2012). For example, nobel metal nanoparticles in colloidal systems ensure uniform dispersion and prevent aggregation, enhancing their optical properties (Li et al., 2016; Anema et al., 2011). By optimizing the colloid’s composition and concentration, SERS signal modulation can be precisely achieved (Xia et al., 2012). Studies show that nanoparticle morphology, concentration, and size distribution significantly influence SERS intensity and reproducibility, highlighting the importance of these parameters for high-sensitivity detection (Herrera et al., 2013; Jiang et al., 2017).

Despite the progress in detecting these traditional analytes, there remains a significant gap in SERS studies concerning other classes of molecules (Xie et al., 2013; Al-Ogaidi et al., 2014). For instance, compounds such as citric acid and those containing carbon-carbon double bonds (C=C) or carbonyl groups (C=O) have received limited attention (Jiang et al., 2017; Cao et al., 2005). Citric acid, a widely occurring organic acid in various biological and environmental systems, presents unique challenges for SERS detection due to its structural characteristics, which differ from those of thiol and amine-containing molecules (Yilmaz and Yilmaz, 2020; Al-Ogaidi et al., 2014).

Moreover, the interaction of SERS substrates with unsaturated organic compounds, such as alkenes and carbonyl-containing compounds, has been less explored, leaving a potential avenue for enhancing the applicability of SERS in diverse fields, including environmental monitoring and food safety (Li et al., 2016). For example, studies on unsaturated fatty acids and aromatic compounds have shown that while they can adsorb onto metallic surfaces, their SERS response is not as well understood or optimized compared to their thiol and amine counterparts.

Here, our research seeks to address this gap by investigating the SERS behavior of Au@Ag core-shell nanoparticles with citric acid and other unsaturated molecules. By examining the Raman peak enhancements in these less-studied analytes, we aim to expand the repertoire of detectable compounds using SERS and to further establish its utility in monitoring applications across environmental, biological, and pharmaceutical domains.

## 2 Results and discussion

### 2.1 SERS enhancement of carboxyl-rich molecules in colloidal

**FIGURE 1:**
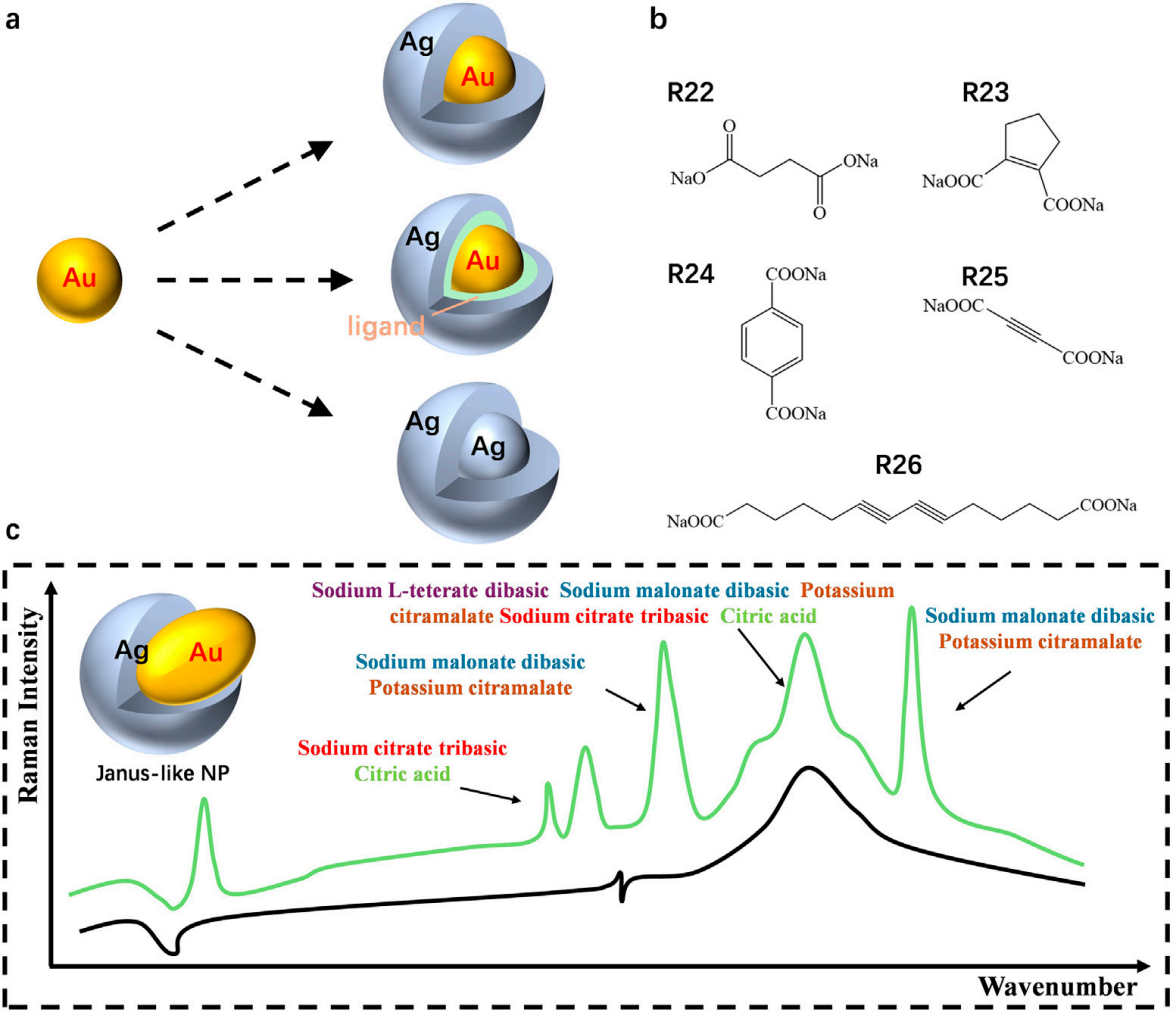
**(A)** Schematics illustrate the surface or interface modification of Au@Ag core-shell nanoparticles. **(B)** Carboxyl-rich molecules being applied in SERS test. **(C)** Raman spectra of orgainic molecules in Au@Ag core-shell nanoparticles colloids.

Figure 1A presents schematic representations of three configurations of the nanoparticle structures: the gold-silver core-shell nanoparticle (Au@Ag), the gold-ligand-silver system (Au@ligand@Ag), and the silver-silver (Ag@Ag) particle configuration (Supplementary Figure S1). These variations highlight the flexibility of modifying the nanoparticle surface for different plasmonic effects by incorporating ligands between the core and shell or using homogeneous metal systems. The interactions between the gold core, silver shell, and intervening ligands play a crucial role in influencing the plasmon resonance properties and SERS performance. Figure 1B shows the molecular structures of several ligands used in the experiments. The ligands include sodium salts of citrate, malonate, and other carboxylate derivatives (e.g., R22–R26), each offering distinct functional groups that affect their binding affinity to metal surfaces. These organic molecules facilitate control over the surface chemistry, optimizing the nanoparticle interface for enhanced plasmonic activity and influencing the nanoparticle aggregation behavior. Figure 1C shows the Raman scattering spectra for different ligands. The spectra reflect the variation in peak intensities and positions as a result of the ligand used, such as sodium citrate, potassium citramalate, and sodium malonate. These differences in peak profiles demonstrate how specific ligand-metal interactions impact the surface plasmon resonance and Raman enhancement. The presence of stronger peaks with specific ligands underlines their potential to fine-tune the nanoparticle’s SERS performance for applications requiring precise molecular detection.

Together, these panels demonstrate the structural and chemical modifications available for optimizing gold-silver core-shell nanoparticles, emphasizing the role of ligands in achieving tunable plasmonic enhancements. This work underpins the importance of controlled synthesis and surface chemistry in improving the sensitivity and selectivity of SERS-based applications.

**FIGURE 2:**
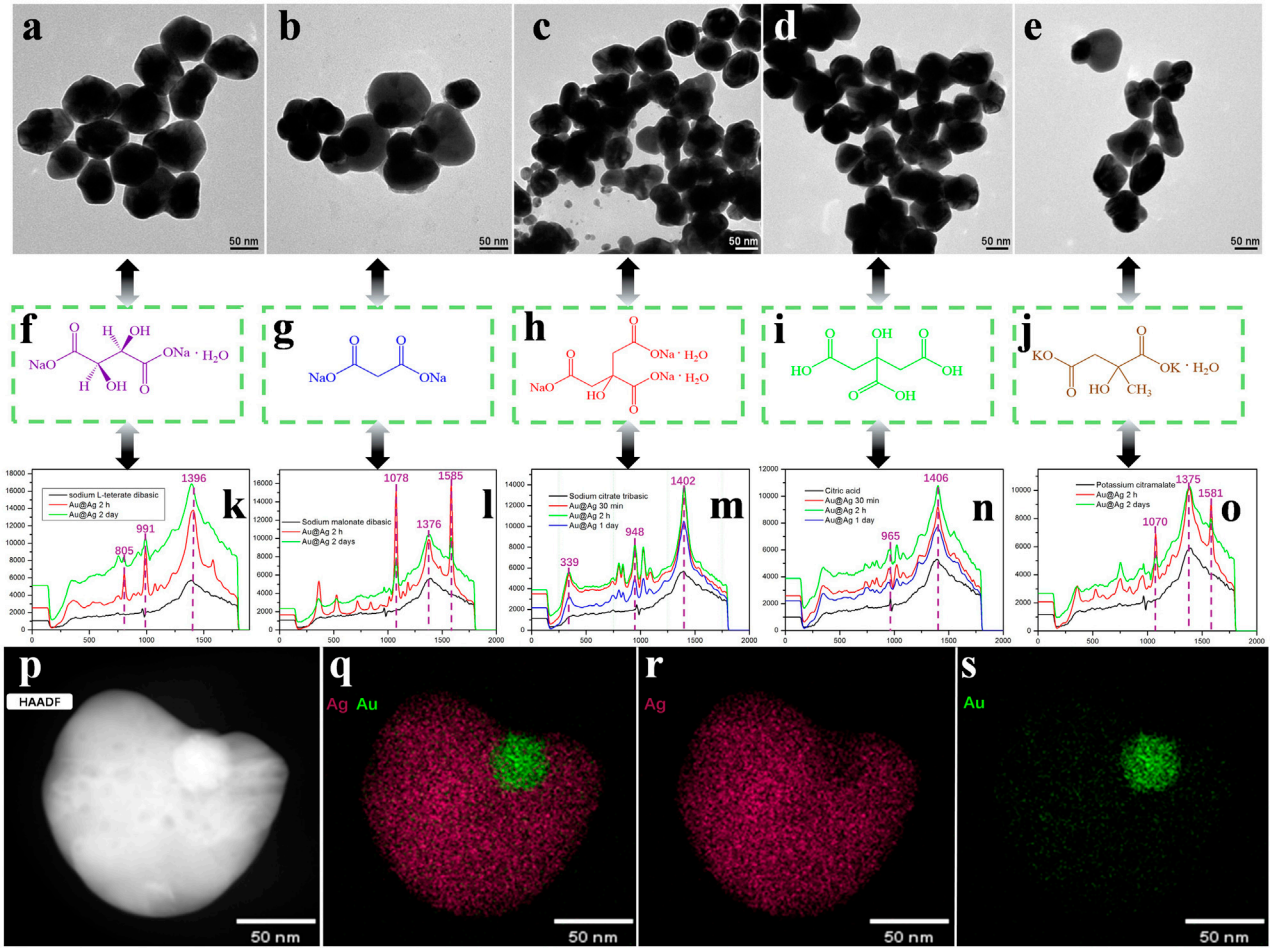
**(A–E)** TEM images of Au@Ag core-shell nanoparticles being applied in colloids **(F–J)** The carboxyl-rich Sodium salts being applied in SERS test **(K–O)** The corresponding Raman spectra of the above samples **(P–S)** STEM image and the EDX color maps showing the core-shell morphology of Au@Ag nanoparticles.

Figure 2 presents a comprehensive examination of the gold-silver core-shell nanoparticles, from the mapping and images provided (presumably in the figures you referenced), it seems that the Au@Ag nanoparticles display a eccentric core-shell shape. A eccentric particle typically has two distinct, often asymmetric faces. In the case of Au@Ag NPs, this could mean that the gold core and silver shell are arranged in a way that results in asymmetric distribution or structural features on opposite sides of the particle (Au@Ag), interacting with different ligands, their corresponding chemical structures, Raman spectra, and EDS mapping results. Figures 2A–E display Transmission Electron Microscopy (TEM) images of the Au@Ag nanoparticles combined with different ligands (Supplementary Figure S10). From left to right, these ligands are Sodium L-tartrate dibasic, Sodium malate, Sodium citrate, Citric acid, and Potassium malate. The images reveal variations in particle size, shape, and dispersion, suggesting that the choice of ligand influences the surface chemistry and aggregation behavior of the nanoparticles. Figures 2F–J illustrates the chemical structures of the corresponding ligands. These structures highlight the presence of carboxylate groups and hydroxyl groups, which play critical roles in stabilizing the nanoparticles and enhancing their plasmonic properties. For example, Sodium L-tartrate and Citric acid contain multiple hydroxyl and carboxylate functionalities, which promote strong interactions with the metallic surfaces, affecting the plasmon resonance characteristics (Otto et al., 2006; Duan et al., 2011; Kudelski, 2005). Figures 2K–O show the Raman spectra of the Au@Ag nanoparticles after being combined with the five ligands. The peak positions of sodium L-tartrate dibasic dihydrate observed in Figure 2K at 805, 991, and 1,386 cm^−1^ can be attributed to specific vibrational modes of the molecule. The peak at 805 cm^−1^ typically corresponds to out-of-plane bending vibrations of the carboxylate group, while the peak at 991 cm^−1^ is often related to C–O stretching vibrations. The peak at 1,386 cm^−1^ is associated with symmetric stretching of the carboxylate ion (–COO⁻). The enhancement of these peaks in SERS can be explained by the increased polarization and resonance effects when the molecules are adsorbed onto the surface of the metallic nanoparticles, which enhances their Raman scattering efficiency. The peak positions of sodium manolate dibasic observed in Figure 2L at 1,078, 1,376, and 1,585 cm^−1^ are related to specific molecular vibrations. The peak at 1,078 cm^−1^ typically corresponds to C–O stretching vibrations, which are common in carboxylate compounds. The 1,376 cm^−1^ peak is often associated with the symmetric stretching of the carboxylate group (–COO⁻). The peak at 1,585 cm^−1^ is usually linked to the aromatic ring vibrations, particularly C=C stretching. The enhancement of these peaks in SERS occurs due to the effective coupling of these vibrational modes with the electromagnetic field generated by the metallic nanoparticles, which amplifies the Raman signal. The peak positions of sodium citrate tribasic observed in Figure 2M at 339, 948, and 1,402 cm^−1^ correspond to specific vibrational modes of the molecule. The peak at 339 cm^−1^ is typically related to out-of-plane bending vibrations, possibly involving the C–C–C or C–O bending modes. The 948 cm^−1^ peak is often associated with C–O stretching vibrations, particularly from the carboxylate groups in the citrate structure. The peak at 1,402 cm^−1^ is usually linked to the symmetric stretching of the carboxylate ions (–COO^−^). The enhancement of these peaks in SERS can be attributed to the resonance and electromagnetic enhancement effects that occur when the sodium citrate molecules are adsorbed onto the surface of metallic nanoparticles, significantly amplifying the Raman signal. The peak positions of citric acid observed in Figure 2N at 965 and 1,406 cm^−1^ correspond to specific vibrational modes of the molecule. The peak at 965 cm^−1^ is generally associated with C–O stretching vibrations, particularly from the hydroxyl and carboxyl groups present in citric acid. The 1,406 cm^−1^ peak is typically linked to the symmetric stretching of the carboxylate groups (–COO^−^). These peaks are enhanced in SERS due to the effective coupling of these vibrational modes with the electromagnetic field generated by metallic nanoparticles, which amplifies the Raman signal, allowing for better detection of citric acid in various applications. The peak positions of potassium citramalate observed in Figure 2O at 1,070, 1,375, and 1,581 cm^−1^ can be attributed to specific vibrational modes of the potassium citramalate molecule. The peak at 1,070 cm⁻^1^ might be associated with C-C or C-O stretching vibrations, which are common in organic compounds. This peak could also involve vibrations of the carboxylate group or other functional groups present in the molecule. The peak at 1,375 cm^−1^ could be related to symmetric or asymmetric stretching of the COO⁻ group, which is a characteristic feature of carboxylic acids and their salts. This vibration is often observed in the fingerprint region of organic compounds in Raman spectroscopy. The peak at 1,581 cm^−1^ might correspond to aromatic ring stretching or other vibrations involving the carbon backbone of the molecule. This peak could also be influenced by the presence of potassium ions and their interaction with the citramalate molecule. It is important to note that the exact assignment of peaks in Raman spectra can be complex and often requires a combination of experimental data and theoretical calculations for accurate interpretation. Additionally, the peak positions can be influenced by factors such as the surface chemistry of the nanoparticles, the orientation of the molecules on the surface, and the experimental conditions used for SERS measurements.

These Surface-Enhanced Raman Scattering (SERS) spectra demonstrate that different ligands significantly impact the intensity and position of the Raman peaks. Sodium citrate and citric acid exhibit notable peak enhancements, indicating their strong influence on the surface plasmon resonance of the Au@Ag nanoparticles. The variations in peak intensities across different samples emphasize the tunability of SERS performance through careful selection of ligands. In addition, we conducted a control experiment without using Au@Ag core-shell structured nanoparticles as the substrate to verify that Au@Ag core-shell structured nanoparticles exhibit SERS enhancement effects when used as the substrate (Supplementary Figures S4–S7).

Figures 2P–S provide Energy Dispersive X-ray Spectroscopy (EDS) mapping results, showing the elemental distribution of Au and Ag within the nanoparticles. Panel (p) displays the HAADF image of a typical Au@Ag nanoparticle, confirming its core-shell structure. Panel (q) presents the merged EDS map, with gold (Au) and silver (Ag) distributions highlighted. Panels (r) and (s) display individual EDS maps for Au and Ag, respectively, confirming the core-shell architecture, where Au is concentrated in the core and Ag forms the outer shell.

The thickness of the silver film significantly impacts SERS signal intensity. Studies show that a 6 nm silver film provides the highest enhancement factor and sensitivity, with good stability (Wu et al., 2022). However, when the thickness exceeds 30 nm, the enhancement factor tends to saturate or decrease due to the blue shift in surface plasmon resonance and reduced hotspot density (Schneidewind et al., 2014). Thin films (less than 20 nm) can increase surface hotspots but may also lead to discontinuity and reduced sensitivity (Lee et al., 2015). Optimal thickness (6–30 nm) balances hotspot density and continuity for the best SERS performance.

Figure 2 demonstrates the profound influence of ligand selection on the structural and plasmonic properties of Au@Ag nanoparticles. The TEM images reveal that ligands affect particle morphology and dispersion, while the SERS spectra highlight the potential to fine-tune plasmonic enhancement through ligand interactions. The EDS mapping further confirms the successful formation of the Au@Ag core-shell structure. These results underline the importance of ligand chemistry in optimizing the performance of plasmonic nanoparticles for SERS-based applications.

### 2.2 Fresh solution or incubated solution?

**FIGURE 3:**
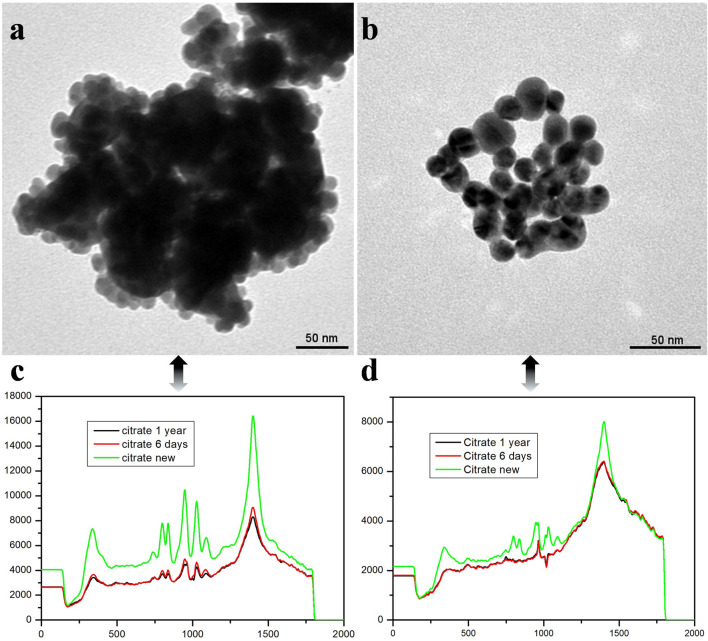
**(A, B)** TEM images of Au@Ag core-shell nanoparticles being applied in colloids. **(C, D)**. The corresponding Raman spectra of the above samples when the tri-sodium citrate salts being applied in SERS test for both fresh and old solutions.

Figure 3A displays the TEM image of Au@Ag nanoparticles synthesized with a 40 nm Au core. These nanoparticles tend to aggregate into denser clusters, suggesting that larger cores promote strong particle-particle interactions. The larger core size creates a more stable platform for silver shell deposition, resulting in smoother surfaces and improved particle packing (Supplementary Figures S2–S3). This aggregation behavior, while enhancing particle coupling, may slightly reduce colloidal stability due to the increased size and density of the clusters.

Figure 3B shows the TEM image of Au@Ag nanoparticles synthesized with a 15 nm Au core. These smaller-core nanoparticles exhibit better dispersion and form more uniform, loosely packed clusters. The increased surface curvature from the smaller core reduces particle aggregation and enhances colloidal stability. However, the thinner silver shell may affect their plasmonic properties, which influences the overall SERS enhancement.

The larger 40 nm gold core generates a stronger localized surface plasmon resonance, leading to an enhanced electromagnetic field at the particle surface. This stronger field amplifies the Raman signals of adsorbed molecules, providing higher SERS sensitivity compared to the weaker field from smaller 15 nm cores (Bhattacharjee et al., 2018b) Besides, the larger core facilitates better plasmon coupling between the gold core and the silver shell, further amplifying the SERS signal. This is less effective in smaller cores due to their limited size (Mei et al., 2020). While the larger 40 nm cores promote stronger particle-particle interactions and result in denser clustering, this aggregation behavior does not necessarily hinder the SERS effect as it contributes to enhanced plasmonic interactions within clusters (Lim et al., 2011). Raman spectra presented in the study show that nanoparticles with 40 nm gold cores consistently exhibit higher peak intensities in SERS compared to those with 15 nm cores. This is attributed to the more effective plasmon resonance and enhanced electromagnetic fields in larger cores (Hu et al., 2016).

Figure 3C and d present the Raman spectra of the Au@Ag nanoparticles with 40 nm and 15 nm Au cores, respectively. In Figure 3, the 40 nm core-based Au@Ag nanoparticles show stronger SERS signals, as indicated by the higher peak intensities in the Raman spectra. The larger gold core facilitates more effective plasmon resonance, enhancing the electromagnetic field at the particle surface. This stronger field amplifies the Raman signals of adsorbed molecules, leading to improved SERS sensitivity.

In contrast, Figure 3D shows that the 15 nm core-based Au@Ag nanoparticles produce weaker SERS signals. Although the smaller core provides some enhancement, the overall electromagnetic field is less intense due to reduced plasmon coupling between the core and shell. The smaller particle size limits the extent of plasmon resonance, resulting in diminished SERS performance compared to the 40 nm core-based particles.

Figure 3 demonstrates that the size of the gold core plays a crucial role in determining both the SERS performance and colloidal stability of Au@Ag nanoparticles. Larger 40 nm Au cores provide stronger plasmonic enhancement, resulting in higher SERS sensitivity but promoting aggregation, which may affect long-term colloidal stability. On the other hand, smaller 15 nm Au cores yield more stable colloids with better dispersion, although their SERS performance is comparatively weaker. These findings highlight the importance of optimizing core size to balance SERS sensitivity and colloidal stability in applications where Au@Ag nanoparticles are employed for molecular detection and sensing.

Overall, the thickness of the silver (Ag) shell in Au@Ag eccentric core-shell nanoparticles plays a crucial role in their Surface-Enhanced Raman Spectroscopy (SERS) performance. Understanding the effects of shell thickness involves examining how the physical properties of the nanoparticle, including surface plasmon resonance, electromagnetic field enhancement, and Raman signal amplification, are influenced by the Ag shell. The finding that a 40 nm silver (Ag) shell on an Au@Ag Janus-like structure results in better SERS (Surface-Enhanced Raman Spectroscopy) performance is intriguing and suggests that the unique structural features of the Janus-like morphology are playing a significant role in the enhanced Raman signal. While a 40 nm Ag shell might seem quite thick compared to conventional core-shell nanoparticles, the Janus structure can offer certain advantages that explain why this thickness works well in our study: i) Janus-like Nanoparticles have an asymmetrical structure where the core-shell composition is not uniform but instead split or arranged in a way that one side (or face) might be significantly different from the other. This asymmetry can give rise to localized surface plasmon resonance (LSPR) phenomena that differ from those in spherical, symmetric nanoparticles. ii) The 40 nm Ag shell on one side of the Janus structure could be creating stronger plasmonic coupling with the environment due to the different dielectric properties between the core (Au) and shell (Ag), especially if the asymmetric configuration creates new or stronger plasmonic “hot spots” at the interfaces between the Au core and Ag shell, or at the junction between the two-halves of the Janus particle. iii) The unique geometry of Janus-like nanoparticles often leads to distinct surface plasmon resonances that are not achievable with spherical or uniform core-shell nanoparticles. The 40 nm Ag shell might enhance the localized electromagnetic field at certain points more effectively in a Janus structure than in a symmetric core-shell nanoparticle, where the signal enhancement could be more uniformly distributed across the nanoparticle’s surface.

Another interesting phenomenon was that the SERS signal is stronger in a fresh solution compared to it in an incubated solution as shown in Raman spectra, regarding less the morphology or size of the nanoparticles. Several factors could be the possible reason: i) Stability of the Citrate Ions: Fresh sodium citrate solutions are likely to have a higher concentration of active citrate ions, which play a crucial role in stabilizing the nanoparticles and preventing agglomeration. Over time, citrate ions may degrade or precipitate, leading to the aggregation of nanoparticles, which can diminish the SERS signal. ii) Surface Charge and Potential: The presence of fresh citrate ions contributes to a higher negative surface charge on the nanoparticles. This charge helps to keep the particles dispersed, enhancing their interaction with target molecules. In older solutions, the reduction in surface charge can lead to increased aggregation, which can result in weaker SERS signals due to reduced active surface area. iii) Chemical Changes in the Solution: As the solution ages, there may be chemical changes, such as the oxidation of citrate or the formation of by-products that could interfere with the SERS activity. These changes can affect the interaction of the probe molecules with the nanoparticles, leading to weaker signals.

### 2.3 Different metal nanoparticles application

**FIGURE 4:**
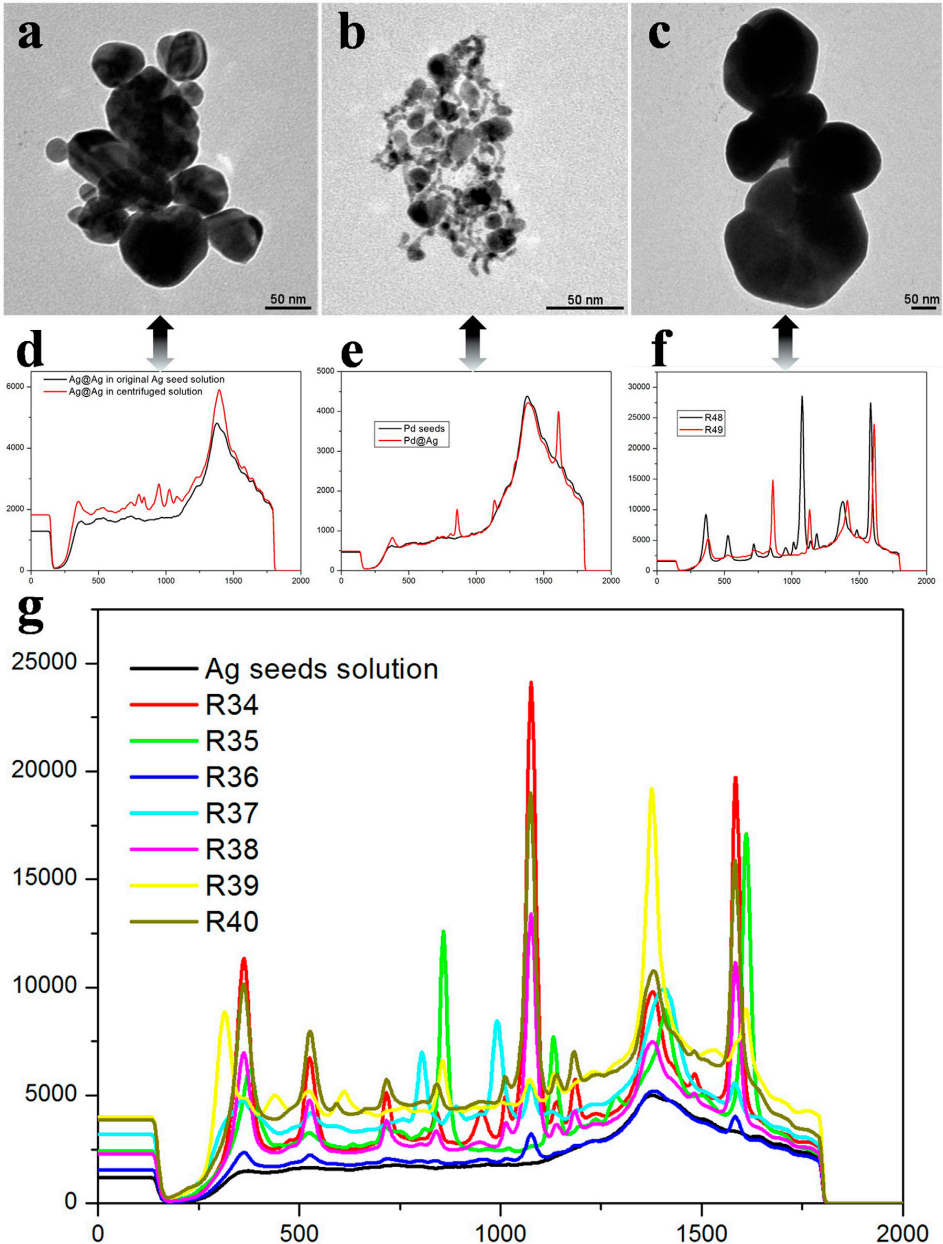
TEM images of **(A)** Ag@Ag, **(B)** Pd@Ag, and **(C)** Ag nanoparticles being applied in colloids. **(D–F)** The corresponding Raman spectra of the above samples when the tri-sodium citrate salts being applied in SERS test for both fresh and old solutions. **(G)** The generality test: different types of carboxyl-rich molecules SERS test in Ag@Ag core-shell nanoparticle solution.

Figure 4 provides an in-depth investigation of the structural properties and SERS performance of three silver-based nanostructures: Ag@Ag, Pd@Ag, and coreless Ag. This comparison evaluates their morphology through TEM images and their plasmonic effects through Raman spectra, along with the impact of different ligands on the SERS performance of Ag@Ag (Supplementary Figures S8–9). Figure 4A presents the TEM image of Ag@Ag nanoparticles. These nanoparticles show a homogenous silver shell over a silver core, which maximizes plasmonic coupling between the core and the shell. This structure is ideal for generating a strong localized surface plasmon resonance (LSPR), crucial for effective SERS enhancement. Figure 4B shows the TEM image of Pd@Ag nanoparticles. The palladium core is coated with a silver shell, offering chemical stability and potential catalytic activity. However, the electron density difference between Pd and Ag may limit plasmonic resonance, making the SERS enhancement less effective compared to the fully silver-based Ag@Ag structure. Figure 4C depicts the TEM image of coreless Ag nanoparticles. These particles exhibit aggregation, with irregular shapes indicating that, without a core-shell structure, their plasmonic field may be less focused. As a result, the absence of a defined core-shell interface reduces the enhancement of the electromagnetic field required for optimal SERS performance. Note: The combination of the Au core and the Ag shell may offer a synergistic effect. The gold core contributes to the stability and electronic properties, while the thicker Ag shell enhances the SERS performance due to silver’s stronger plasmonic properties at visible wavelengths.

Figure 4D displays the SERS spectrum of Ag@Ag nanoparticles. The high intensity and distinct Raman peaks demonstrate the excellent SERS enhancement provided by the Ag@Ag structure, driven by the strong plasmonic coupling between the core and the shell. Figure 4E presents the SERS spectrum of Pd@Ag nanoparticles. Although the silver shell contributes to the SERS signal, the presence of a palladium core introduces limitations to the plasmonic field, leading to moderate SERS enhancement compared to Ag@Ag. Figure 4F shows the SERS spectrum of coreless Ag nanoparticles. The absence of a core-shell configuration reduces the overall electromagnetic field enhancement, resulting in the weakest SERS performance among the three structures.

To test the generality of our findings, a series of carboxyl-rich molecules have been applied in SERS test. As shown in Figure 4G, nearly all organic molecules rich in carboxyl groups exhibited SERS enhancement, particularly at the characteristic peaks of 362 cm^−1^, 1,081 cm^−1^, and 1,597 cm^−1^, where the enhancement was even more pronounced. These results indicate that precise measurements can be made for carboxyl-rich organic molecules, with signal intensities increasing exponentially. This has promising applications in fields such as biological monitoring and food safety. It is notably that the thickness of the Ag shell on Au@Ag core-shell nanoparticles is a key factor in determining how well these particles enhance the SERS signal. This is because the shell thickness influences several aspects of the nanoparticle’s plasmonic behavior, including field enhancement, hot spot formation, and analyte-molecule interactions. In this case, the 40 nm Ag shell in the Janus-like Au@Ag nanoparticle structure likely benefits from a combination of factors, including its ability to generate strong localized fields at the interfaces, coupled with the unique shape or asymmetry of the Janus structure, leading to better SERS performance than what would be expected from traditional, more symmetrical core-shell nanoparticles.

Figure 4 demonstrates that Ag@Ag nanoparticles deliver the best SERS enhancement due to strong plasmonic coupling between the core and shell. Pd@Ag nanoparticles provide moderate enhancement, with the palladium core reducing the plasmonic effect but offering chemical stability. Coreless Ag nanoparticles exhibit the weakest SERS performance, highlighting the importance of core-shell structures for efficient electromagnetic field amplification. Additionally, the analysis emphasizes that ligand selection plays a crucial role in fine-tuning SERS sensitivity by modulating surface interactions. These findings highlight the need for optimizing material composition, structural design, and surface chemistry to achieve high-performance SERS platforms for sensing applications. We verified that our designed Au@Ag core-shell nanostructures can effectively enhance Raman signal intensity by calculating the SERS performance factor (SPF) and enhancement factor (EF) based on the literature (Litti and Meneghetti, 2019; Yue et al., 2024; Fales et al., 2011).

## 3 Conclusion

This study demonstrates the significant potential of gold-silver core-shell nanoparticles (Au@Ag) in enhancing Surface-Enhanced Raman Scattering (SERS) signals of carboxyl-rich molecules in colloidal. Through a comprehensive analysis of different structural configurations, core sizes, and ligand interactions, it was established that Au@Ag nanoparticles offer optimal SERS performance due to the strong plasmonic coupling between the gold core and silver shell. The incorporation of silver on a stable gold core provides enhanced Raman signal amplification of carboxyl-rich molecules without -SH, or -NH_2_ groups while improving chemical stability and biocompatibility compared to pure silver nanoparticles. Additionally, the experiments reveal that the size of the gold core plays a pivotal role in balancing SERS sensitivity and colloidal stability, with larger cores yielding higher SERS signals but lower stability. In contrast, smaller cores exhibit better dispersion but weaker enhancement. Furthermore, the choice of ligands significantly influences SERS performance, as specific ligand-metal interactions optimize the plasmon resonance effect, further enhancing signal intensity and sensitivity. This work provides valuable insights into the design and optimization of bimetallic core-shell nanostructures for molecular detection and sensing.

## Data Availability

The original contributions presented in the study are included in the article/Supplementary Material, further inquiries can be directed to the corresponding author.
