# Supplementary material for: Investigating Raman peak enhancement in carboxyl-rich molecules: insights from Au@Ag core-shell nanoparticles in colloids
Source: Front Chem. 2025 Jan 27;13:1522043. doi: 10.3389/fchem.2025.1522043 (PMC11808131; doi:10.3389/fchem.2025.1522043)
Supplement: Supplementary file 1 [file DataSheet1.docx]

**Investigating Raman Peak Enhancement in Carboxyl-Rich Molecules: Insights from Au@Ag Core-Shell Nanoparticles in Colloids**

*Junhao Chen*^1^, *Zhengjia Chen*^2^*, Tong Liang*^1^, *Zhennan Zhang*^1^*, Dahang Cheng*^1^*, Shurui Liu*^1^*, Haiyang Liu*^1^*,* Cuicui Liu^3^, *Xiaohui Song*^1,*^

^1^School of Materials Science and Engineering, Hefei University of Technology, Anhui Province, 230009, China

^2^Chongqing Polytechnic University of Electronic Technology, Chongqing, 401331, China

^3^Department of Chemistry and Biochemistry, Nanyang Technological University, Singapore 637616, Singapore

* To whom correspondence may be addressed: xiaohuisong@hfut.edu.cn

**Content:**

Experimental

Supporting Figures S1-10

Supporting tables 1

**Experimental**

**Synthesis of Au@Ag core-shell nanoparticles with different diameters of gold nanoparticles:** First, weigh 5 mg of solid AgNO₃ particles and dissolve them in 10 mL of distilled water. Stir the solution with a magnetic stirrer for 10 minutes to obtain a 5 mg/mL AgNO₃ aqueous solution. Next, weigh 10 mg of ascorbic acid solid and dissolve it in 10 mL of distilled water. Stir it with a magnetic stirrer for 10 minutes to obtain a 5 mg/mL ascorbic acid solution.Then, take 2 mL of a 40 nm gold nanoparticle solution and add it to a centrifuge tube, followed by 10 mL of distilled water. Centrifuge the solution at 8,000 rpm for 5 minutes. After centrifugation, discard the supernatant and add 10 mL of distilled water again, then repeat the centrifugation under the same conditions (8,000 rpm for 5 minutes). Remove the supernatant and add 1 mL of distilled water to obtain a purified 40 nm gold nanoparticle aqueous solution without CTAB. To the purified 40 nm gold nanoparticle solution, add 2 mL of the prepared 5 mg/mL AgNO₃ aqueous solution, followed by 0.2 mL of the 5 mg/mL ascorbic acid solution. Observe the solution change color from red to bluish-gray, indicating the formation of Au@Ag nanoparticles combined with ligands. The same procedure can be applied to a 15 nm gold nanoparticle solution to obtain Au@Ag nanoparticles with a 15 nm gold core.

**Supporting Figures S1-S10**


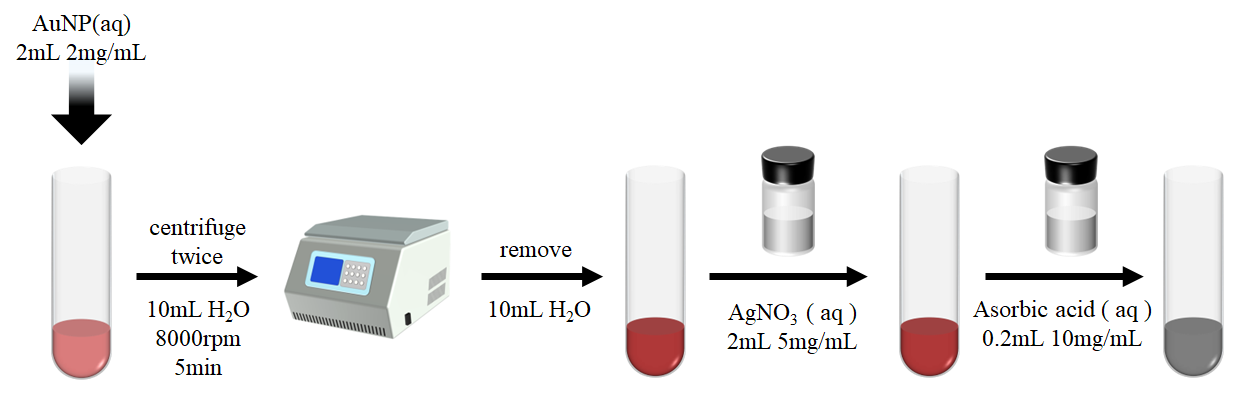


**Fig. S1** Flow chart of synthesis experiment , separation and purification of Au@AG nanoparticles.


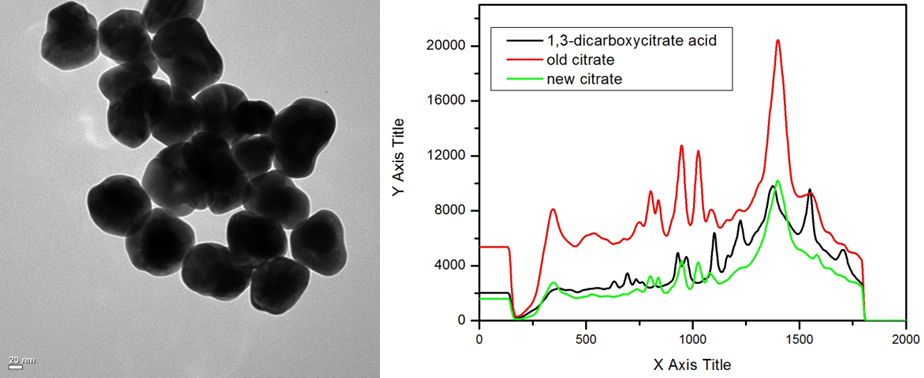


**Fig. S2** TEM images and Raman spectra of 60 nm Au synthesized Au@Ag core-shell structured nanoparticles with different ligands.


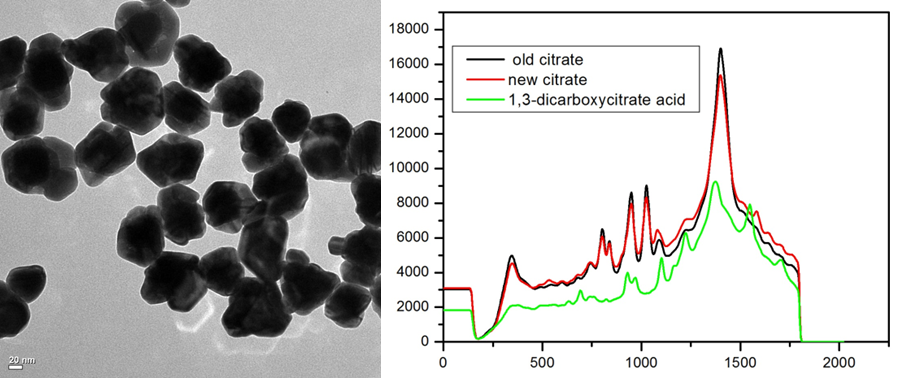


**Fig. S3** TEM images and Raman spectra of 60 nm Au synthesized Au@Ag core-shell structured nanoparticles with different ligands in the hydroquinone system.


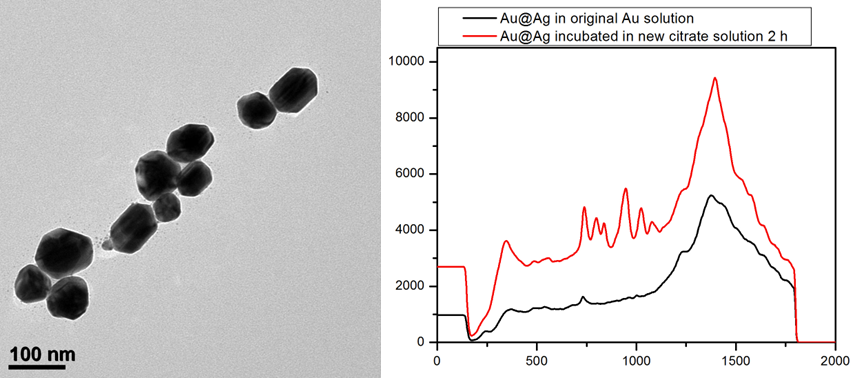


**Fig. S4** TEM and Raman spectra of Au original solution, followed by Au@Ag synthesis, with Au@Ag centrifuged and redispersed into new citrate solution for 2-hour incubation.


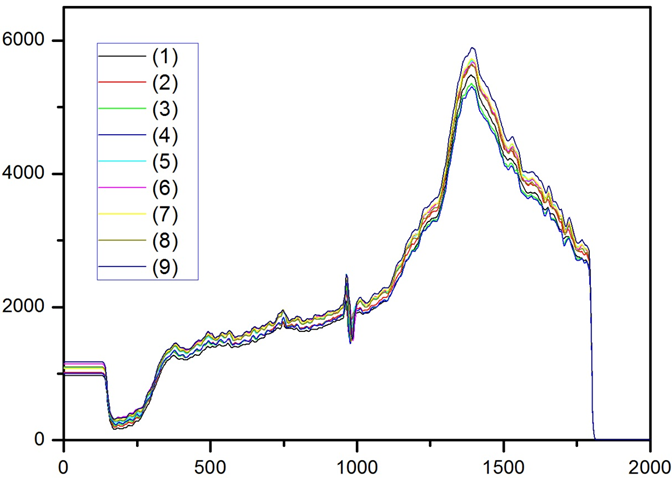


**Fig. S5** The chemical names of the ligands used. (1) sodium citrate 1 year, (2) sodium citrate 6 days, (3) sodium citrate new, (4) citric acid, (5) 1,3-dicarboxycitric acid, (6) sodium citrate tribasic, (7) sodium L-tartrate dibasic, (8) sodium malonate dibasic, (9) potassium citrate malonate.


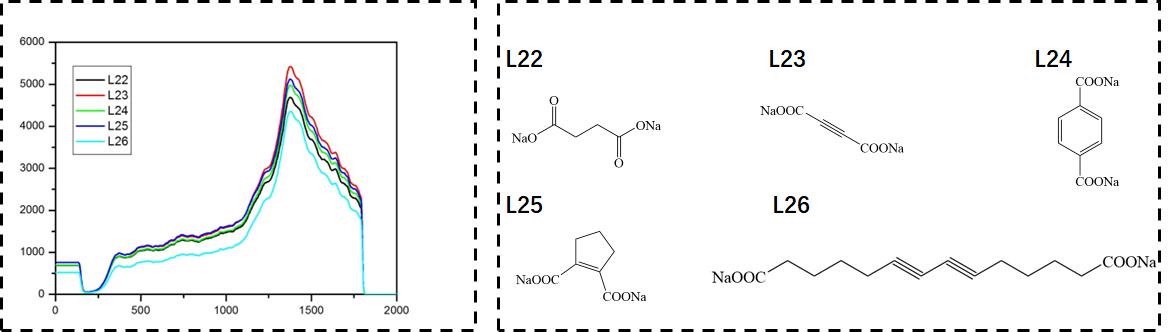


**Fig. S6** Raman spectra of ligands without using Au@Ag core-shell structured nanoparticles as the substrate.


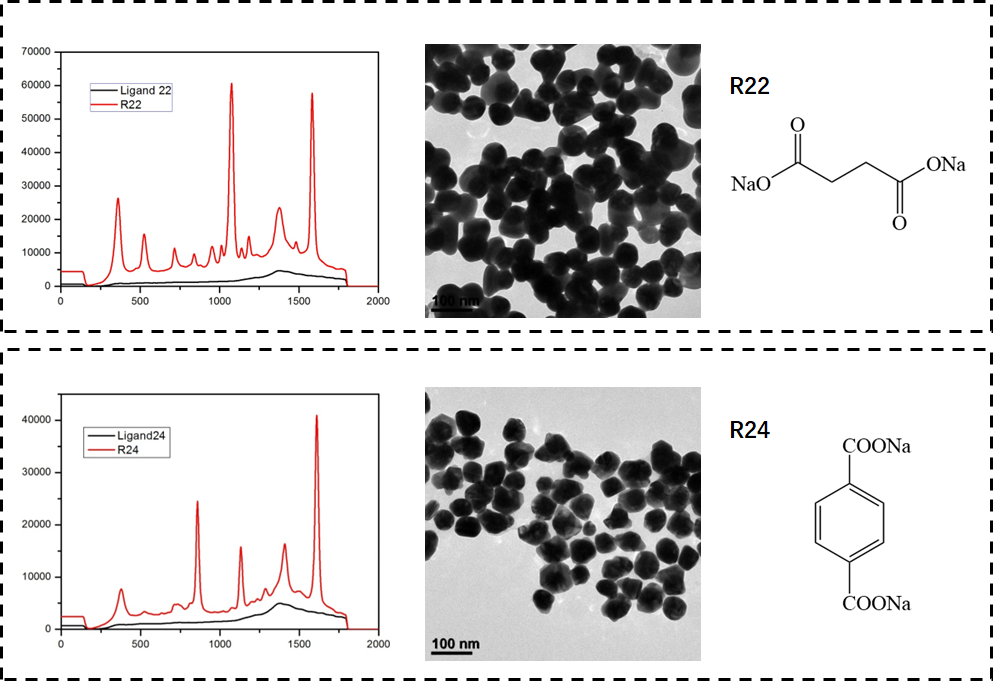


**Fig. S7** Raman spectra, TEM images, and chemical structures of the ligands R22 and R24 using Au@Ag core-shell structured nanoparticles as the substrate.


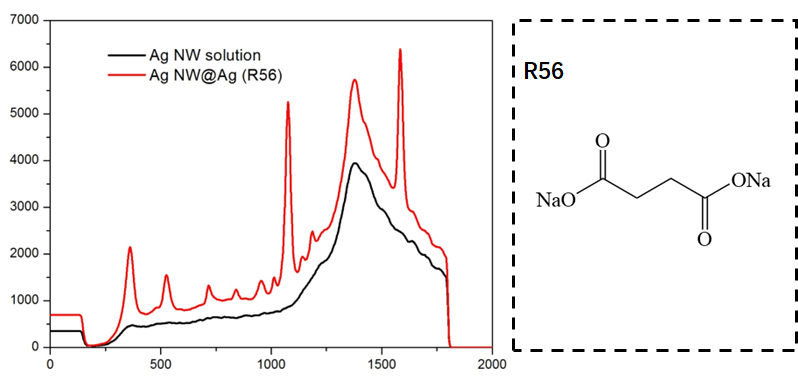


**Fig. S8** SERS-enhanced Raman spectra using Ag nanowires and Ag nanowires@Ag for the R54 ligand, along with the chemical structure of R54.


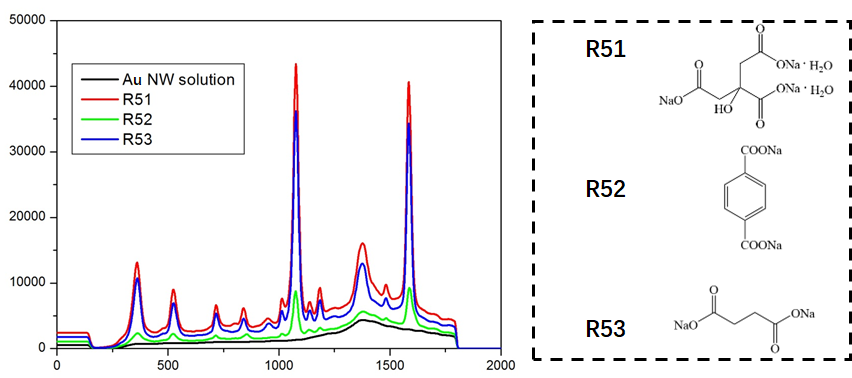


**Fig. S9** Raman spectra of gold nanowires and SERS-enhanced Raman spectra of R51, R52, and R53 using Au nanowires as the substrate, along with their chemical structures.


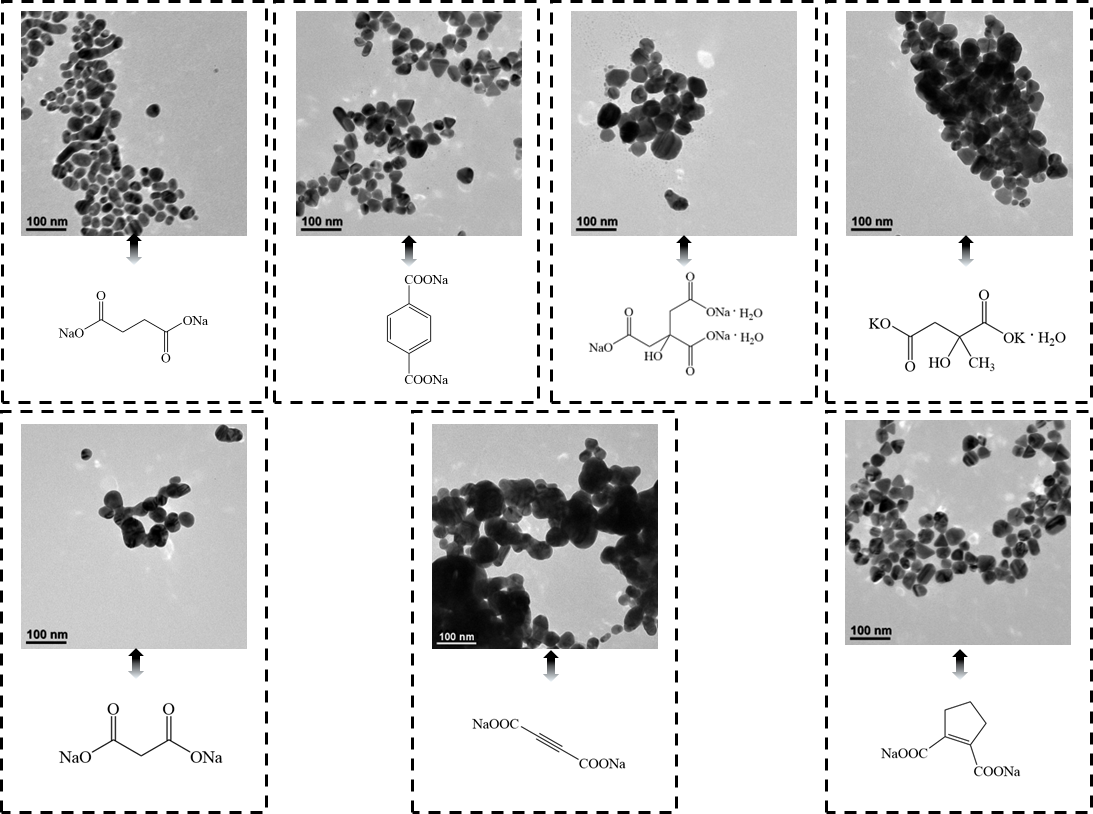


**Fig. S10** TEM images of different ligands synthesized with Au@Ag core-shell structured nanoparticles and their corresponding chemical structures.

**Supplementary Tables**

**Table 1**. Ligands combined with Au@Ag in the experiments

| **Abbreviations** | **Full name** | **Chemicals** |
| --- | --- | --- |
| R22 | sodium malate | 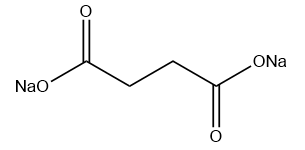 |
| R23 | sodium citrate | 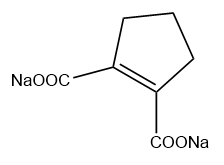 |
| R24 | sodium isophthalate | 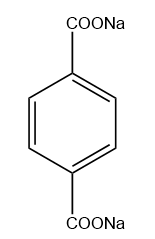 |
| R25 | disodium butynedioate | 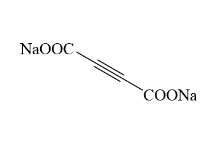 |
| R26 | disodium linoleate | 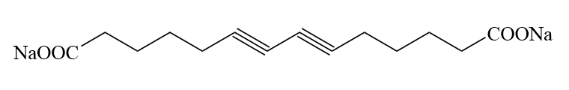 |
